# Identification of small marker chromosomes using microarray comparative genomic hybridization and multicolor fluorescent *in situ* hybridization

**DOI:** 10.1186/s13039-016-0273-5

**Published:** 2016-08-08

**Authors:** Woori Jang, Hyojin Chae, Jiyeon Kim, Jung-Ok Son, Seok Chan Kim, Bo Kyung Koo, Myungshin Kim, Yonggoo Kim, In Yang Park, In Kyung Sung

**Affiliations:** 1 Department of Laboratory Medicine, College of Medicine, The Catholic University of Korea, Seoul, Korea; 2 Catholic Genetic Laboratory Center, Seoul St. Mary’s Hospital, College of Medicine, The Catholic University of Korea, Seoul, Korea; 3 Department of Obstetrics and Gynecology, College of Medicine, The Catholic University of Korea, Seoul, Korea; 4 Department of Pediatrics, College of Medicine, The Catholic University of Korea, Seoul, Korea; 5 Department of Laboratory Medicine, Seoul St. Mary’s Hospital, College of Medicine, The Catholic University of Korea, 222 Banpo-daero, Seocho-gu, Seoul, 137-701 Korea

**Keywords:** Marker chromosome, Array comparative genomic hybridization, Fluorescence *in situ* hybridization

## Abstract

**Background:**

Marker chromosomes are small supernumerary chromosomes that cannot be unambiguously identified by chromosome banding techniques alone. However, the precise characterization of marker chromosomes is important for prenatal diagnosis and proper genetic counseling. In this study, we evaluated the chromosomal origin of marker chromosomes using a combination of banding cytogenetics and molecular cytogenetic techniques including diverse fluorescence *in situ* hybridization (FISH) assays and array comparative genomic hybridization (array CGH).

**Results:**

In a series of 2871 patients for whom cytogenetic analysis was requested, 14 cases with small supernumerary marker chromosomes (sSMCs) were identified. Nine sSMCs were mosaic, and five nonmosaic. Of the nine cases with known parental origins, four were identified as de novo, and four and one were maternally and paternally inherited, respectively. Six sSMCs were identified by FISH using centromeric probes; three sSMCs were derived from chromosome 15, including two heterochromatic sSMC(15)s and a large sSMC(15) spanning 15q11.1q13.1, and three sSMCs originated from chromosome 14 or 22. Array CGH revealed two cases with derivatives of chromosome 2 and whole chromosome painting multicolor-FISH (M-FISH) identified three cases with derivatives of chromosome 6, 16, and 19, respectively. One maker chromosome in Turner syndrome was characterized as sSMC(X) by preferential application of a centromeric probe for X-chromosome. In addition, one sSMC composed of genomic materials from chromosomes 12 and 18 was identified in parallel with parental karyotype analysis that revealed the reciprocal balanced translocation.

**Conclusions:**

This report is the largest study on sSMCs in Korea and expands the spectrum of sSMCs that are molecularly characterized.

**Electronic supplementary material:**

The online version of this article (doi:10.1186/s13039-016-0273-5) contains supplementary material, which is available to authorized users.

## Background

Marker chromosomes, also known as small supernumerary marker chromosomes (sSMCs), are structurally abnormal chromosomes that cannot be unambiguously identified or characterized by conventional banding cytogenetics (ISCN 2013) [1]. They are generally equal or smaller in size than a chromosome 20 of the same metaphase spread [2], and the small size of markers precludes the identification of their chromosomal origin by conventional banding techniques, and molecular cytogenetic techniques are necessary for their characterization.

According to a recent, comprehensive review [3], marker chromosomes are found in 0.075 % of unselected prenatal cases, and in 0.044 % of consecutive postnatal cases, but frequencies are elevated to 0.125 % in infertile subjects and to 0.255 % in developmentally retarded patients [3]. In terms of the parental origin of marker chromosomes, approximately 30 % of markers are familial, while 70 % are *de novo*. The clinical phenotypes associated with marker chromosomes are also highly variable, from normal to severely abnormal [4], and this renders marker chromosomes a particularly difficult problem in genetic counseling, especially in prenatal *de novo* cases. There has been a previous report on marker chromosomes identified in Korean patients [5] investigated with fluorescence *in situ* hybridization (FISH) analysis, but with advancements in molecular cytogenetic diagnostics, tools including whole-chromosome painting FISH and array comparative genomic hybridization (array CGH) have been applied in characterization of marker chromosomes.

Therefore, in this study, we aimed to characterize consecutive marker chromosomes identified from a single genetic center in Korea, with multiple molecular cytogenetic methods in combination with banding cytogenetics, to accurately characterize the chromosomal origin and the genetic content of marker chromosomes.

## Methods

Chromosomal analysis, referred for constitutional abnormality, was performed on 2871 patients (1974 peripheral blood specimens, 897 amniotic fluid specimens) from January 2010 to December 2013 at Seoul St. Mary’s Hospital. Written informed consent was obtained from the patients and/or their family members. Whenever available, the familial occurrence of markers was evaluated through parental studies. Information on the phenotypic features of the patients was obtained by a review of medical records. This study was conducted in accordance with the ethical guidelines of the Declaration of Helsinki and was approved by the Institutional Review Board (IRB)/Ethics Committee of Seoul St. Mary’s Hospital (IRB No.KC11TISI0277).

### Banding cytogenetics

Banding cytogenetics was performed on G-banded metaphase chromosomes of cultured peripheral blood lymphocytes and/or amniotic fluid cells using routine techniques. Karyotypes were interpreted according to the ISCN 2013.

### FISH studies

If the size of the marker chromosome is similar to a chromosome 20 of the same metaphase spread, FISH analysis using centromeric probes for chromosomes 15, 18, and 12 was performed. If the size of the marker is smaller than a chromosome 20, FISH analysis using centromeric probes for all acrocentric chromosomes 15, 13/21, and 14/22 was performed. In Turner syndrome (TS) patients with a marker chromosome (45,X/46,X,+mar), FISH studies using X centromeric probe as well as SRY were performed. And in all patients with marker chromosomes, parental study was performed in parallel, whenever possible. The FISH probes used in this study are summarized in Additional file 1: Table S1.

If the origin of the marker chromosome was not clarified by the above strategies, we then performed whole chromosome painting multicolor-FISH (M-FISH) and/or array CGH, based on the level of mosaicism, and the amount of specimen available. M-FISH was performed using the 24 *X*Cyte Human Multicolor FISH Probe kit (MetaSystems, Altlussheim, Germany) according to the manufacturer’s instructions. Fluorescent images were captured and analysed using an Axio Imager 2 fluorescence microscope (Zeiss, Jena, Germany) and Isis image analysis software (MetaSystems).

### Array CGH

Array CGH analysis was performed using a SurePrint G3 Human CGH Microarray 8 X 60 K kit (Agilent Technologies, Santa Clara, CA, USA), which consisted of 62,976 oligonucleotide probes spaced at 41 kbp intervals (median probe spacing) throughout the genome. Control DNA (Promega Corp., Nepean, Canada) was used as the reference DNA. DNA digestion, labeling and hybridization were performed following the manufacturer’s instructions. Scanned images were quantified using Agilent Feature Extraction software (v10.0), and the resulting data were imported into Agilent Genomic Workbench 7.0.4.0 software for visualization, and copy number variations were detected using the Aberration Detection Method-2 (ADM-2) algorithm. All genomic coordinates were based on human genome build hg19/GRCh37.

## Results

Of 2871 patients referred for chromosomal analysis, marker chromosomes were identified in 14 patients. Parental study was performed in nine patients, and five marker chromosomes (55.6 %) were inherited from one of the parents, while four markers (44.4 %) were *de novo*. Of the five inherited markers, four (80 %) were maternally inherited and only one (20 %) was paternally inherited. Mosaicism was detected in nine patients (64 %), whereas a single cell line was observed in the remaining five patients (36 %). Three marker chromosomes (21 %) were equal in size to a chromosome 20, whereas the other 11 (79 %) were smaller than a chromosome 20 of the same metaphase spread. Although the depth of clinical information available differed among the subjects, most postnatal cases (10/11, 91 %) showed abnormal phenotypes of variable severity (Table 1). All three prenatal cases were referred for advanced maternal age, and in two cases parental study was available, and one was maternally inherited and one *de novo*, and the outcome of the pregnancies could not be followed up.

**Table 1:** Summary of fourteen cases showing marker chromosomes

Case No.	Gender	Age at testing	Karyotype	Chr. Origin	Size	Inheritance	Phenotype	Molecular cytogenetic method	Molecular cytogenetic method results	Fig
1	female	19 y	mos 45,X[25]/46,X,+mar[5]	X	<20	NE	Short stature, Primary amenorrhea	Centromeric FISH	nuc ish(DXZ1x1)[340/400]/nuc ish(DXZ1x2)[60/400]	Fig. 1
2	female	1 m	47,XX,+der(12;18)(p10;p10)	der(12;18)(p10;p10)	<20	paternal	Incomplete cleft palate,	M-FISH	ish der(12;18)(wcp12+,wcp18+) pat	Fig. 2
							Developmental coordination disorder,			
							Developmental delay			
3	female	25 m	mos 47,XX,+mar[24]/46,XX[6]	15	~20	NE	Developmental delay, Failure to thrive	Centromeric FISH	ish idic(15)(D15Z1++, D15S11-)	Fig. 3a, 3b
4	male	prenatal	47,XY,+mar	15	~20	maternal	NA	Centromeric FISH, M-FISH	ish idic(15)(wcp15+, D15Z1++, D15S11-) mat	Fig. 3c, 3d
5	female	32 m	47,XX,+mar	15	~20	maternal	Developmental delay	Centromeric FISH, Array CGH	ish der(15)(D15Z1+).arr[hg19]15q11.1q13.1(20,102,541-28,525,460)x3 mat	Fig. 3e
6	male	prenatal	mos 47,XY,+mar[14]/46,XY[26]	14	<20	NE	NA	Centromeric FISH, M-FISH	ish der(14)(D14Z1+,wcp14+)	Fig. 4a
7	NA	NA	NA	22	<20	NE	NA	Centromeric FISH, M-FISH	ish idic(22)(D22Z1++, wcp22+)	Fig. 4b
8	female	11 y	mos 47,XX,+mar[45]/46,XX[5]	14 or 22	<20	maternal	Short stature	Centromeric FISH, M-FISH	ish der(14/22)(D14Z1/D22Z1+) mat	Fig. 4c
9	female	prenatal	mos 46,XX,min[4]/46,XX[11]	6	<20	de novo	NA	Centromeric FISH, M-FISH	ish der(6)(wcp6+) de novo	NA
10	female	8 y	mos 47,XX,+mar[5]/46,XY[45]	16	<20	NE	Short stature,	M-FISH	ish der(16)(wcp16+)	Fig. 7a
							Elevated TSH level			
11	male	18 m	mos 47,XY,+mar[14]/46,XY[26]	19	<20	de novo	Developmental delay	M-FISH	ish der(19)(wcp19+) de novo	Fig. 7b
12	female	10 y	47,XX,+mar	2	<20	maternal	Short stature, Developmental delay	Array CGH	arr[hg19]2q11.1q12.3(95,529,039-108,083,956)x3 mat,18p11.32p11.31(142,096-5,853,122)x1 dn	Fig. 5a
13	male	2 m	mos 47,XY,+mar[8]/46,XY[42]	2	<20	de novo	Prematurity, Developmental delay, ASD	Array CGH, FISH	arr[hg19]2q11.1q12.1(95,529,039-105,358,887)x3 dn,7q11.23(72,726,578-74,139,390)x3 mat	Fig. 5c,
										Fig. 6
14	male	20 m	mos 48,XY,+2mar[16]/47,XY,+mar[9]/46,XY[5]	NE	<20	de novo	Developmental delay	NE	NE	NE

*Chr* chromosomal, *CGH* comparative genomic hybridization, *M-FISH* whole chromosome painting multicolor- fluorescence *in situ* hybridization, *NA* not available, *NE* not established

In the present study, chromosomal origins were identified in 13 of 14 identified marker chromosomes, and in one case, further characterization was not possible because there was not enough material. These marker chromosomes originated from various chromosomes and consisted of three cases (23 %) with derivatives of chromosome 15, two cases (15 %) with derivatives of chromosome 2, and one case (8 %) each with derivatives of chromosome 6, 14, 16, 19, 22, 14/22 and der(12;18)(p10;p10). Also, in a patient with TS (45,X/46,X,+mar), the marker chromosome originated from the X chromosome.

**Fig. 1:**
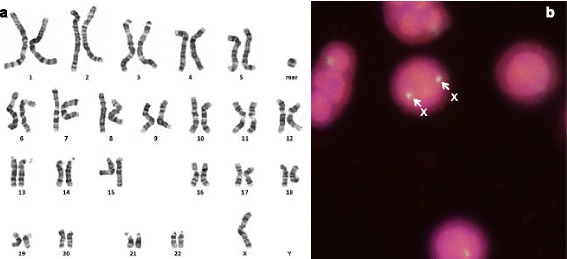
**a** Karyotype of the patient with Turner syndrome; **b** Interphase FISH analysis using the probes of DXZ (Xp11.1-q11.1; spectrum green) and SRY (Yp11.31; spectrum red) probes shows two copies of green and absence of red signals

**Fig. 2:**
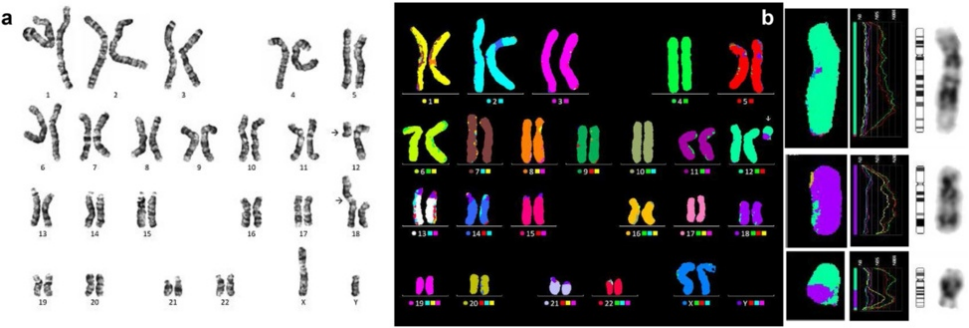
**a** Karyotype of the patient’s father showing 46,XY,t(12;18)(p10;p10); **b** M-FISH analysis demonstrated that sSMC was structured from chromosome 12 and 18 in case 2

In accordance with the presented approach for marker chromosome characterization, preferential application of a centromeric probe specific for X- and Y-chromosome identified the marker as mar(X) in a TS patient (Fig. 1). And for an inherited marker chromosome (case 2), the identification of a balanced translocation in one of the parents led to a straightforward characterization of the marker chromosome as 47,XX,+der(12;18)(p10;p10) (Fig. 2).

**Fig. 3:**
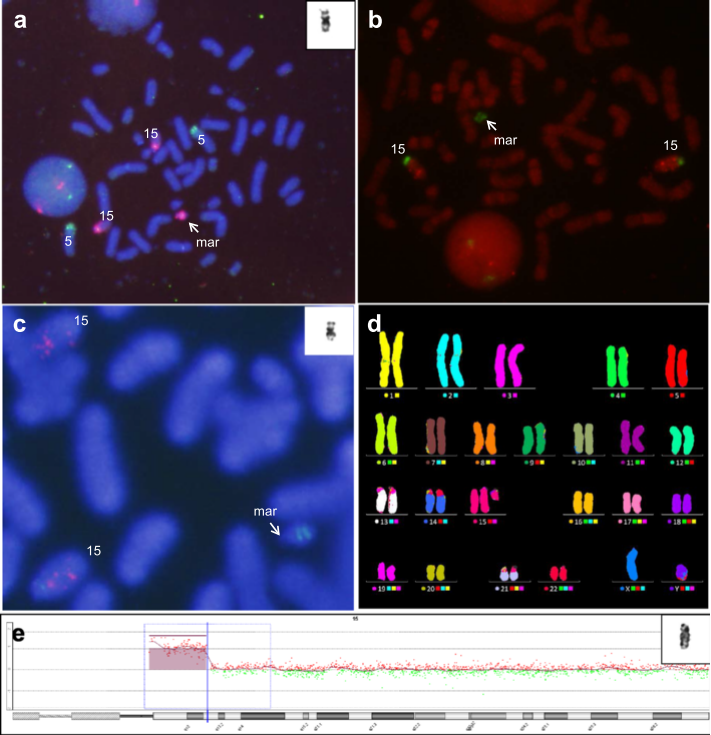
Identification of the chromosomal origin of the sSMC(15) by FISH and array CGH. **a** FISH analysis using D15Z1 (15p11.2; spectrum red) probe shows a red hybridization signal on the marker chromosome (case 3); **b**-**c** FISH analysis of the idic(15) using D15Z1 (15p11.2; spectrum green) and D15S11 (15q11-q13; spectrum red). sSMCs have two green hybridization signals of D15Z1 with no D15S11 signal (case 3, B; case 4, C); **d** SMC(15) identified with M-FISH 15 (case 4); **e** Array CGH analysis results showing amplification from 15q11.1 to 15q13.1 (chr15: 20,102,541-28,525,460), spanning 8.4 Mb (case 5)

**Fig. 4:**
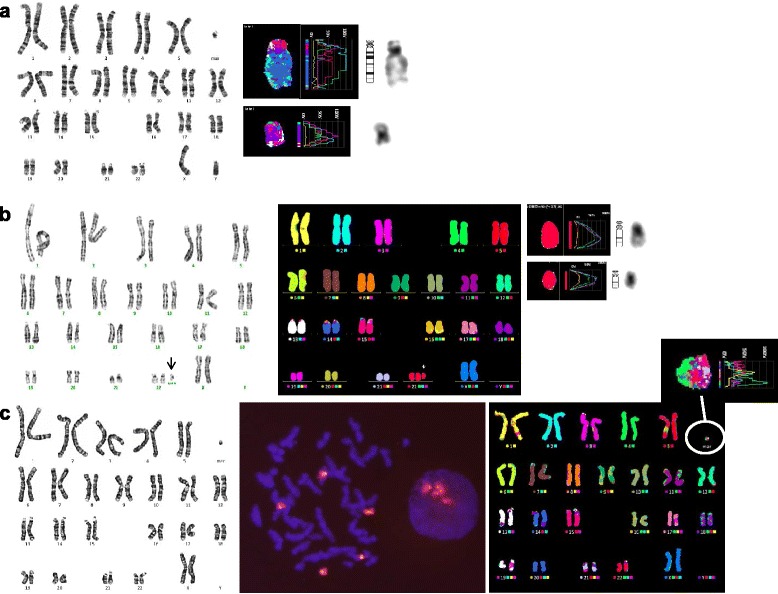
M-FISH analysis profiles of sSMC(14) or sSMC(22). **a** The marker contained chromosome 14 material (case 6); **b** The dicentric marker contained chromosome 22 material (case 7); **c** FISH analysis of the sSMC using an alpha satellite probe D14Z1/D22Z1, cep14/22 (spectrum red) showing positive hybridization signals on two chromosomes 14, two chromosomes 22 and the marker chromosome. M-FISH could not distinguish between chromosome 14 and chromosome 22 (case 8)

The three marker chromosomes equal in size to a chromosome 20 (cases 3, 4 and 5) were all derived from chromosome 15, and this was readily identified by FISH analysis using centromeric probes for chromosome 15. FISH with centromeric probes for chromosome 12, and 18 was also performed to identify i(12p) associated with Pallister-Killian (OMIM 601803) syndrome and i(18p) syndrome, respectively, but no i(12p) nor i(18p) was found in this study. For both sSMC(15)s of case 3 and 4, FISH analysis using probe D15S11(15q11.2) lacked a positive hybridization signal and were therefore considered as heterochromatic (Fig. 3a-d). However, in sSMC(15) of case 5, array CGH showed a 8.4 Mb gain of chromosome 15q11.1q13.1 (chr15:20,102,541-28,525,460) encompassing *ATP10A*, *CYFIP1*, *GABRA5*, *GABRB3*, *GABRG3*, *HERC2*, *MAGEL2, MKRN3, NDN*, *NIPA1*, *NIPA2*, *OCA2*, *POTEB*, *SNRPN*, *TUBGCP5,* and *UBE3A* genes, with a log_2_ ratio of 1.0409, inherited from a phenotypically normal mother (Fig. 3e). For the marker chromosomes smaller than that of a chromosome 20, FISH analysis using centromeric probes for all acrocentric chromosomes was performed sequentially according to their reported frequency in the literature, starting with chromosome 15, followed by 14/22 and 13/21. This led to the characterization of three cases that originated from chromosome 14 and/or 22 (Fig. 4). Therefore, in a total of six cases, the chromosomal origin of the marker chromosome was ascertained by FISH probes targeting the centromere.

**Fig. 5:**
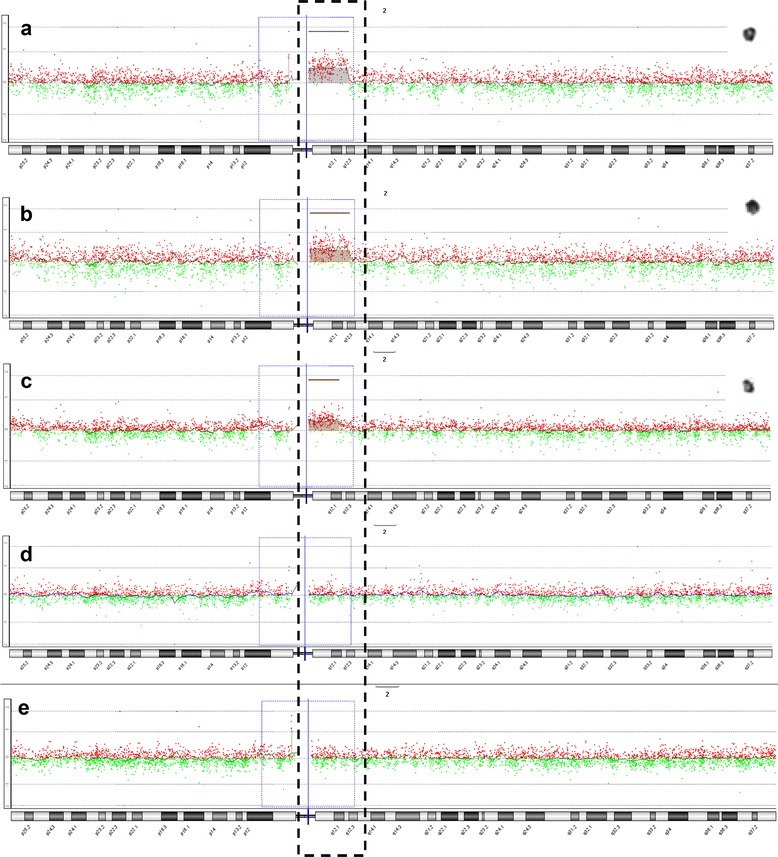
Array CGH analysis profiles of sSMC(2)s. **a** Array CGH detected a gain of 12.6 Mb derived from chromosome 2q11.1q12.3 (chr2:95,529,039-108,083,956) (case 12); **b** Identical duplication shown in her mother; **c** De novo 9.8 Mb gain of chromosome 2q11.1q12.1 (chr2: 95,529,039-105,358,887) (case 13). **d**-**e** Genomic microarray ratio plots for chromosome 2 showing no imbalances in both father (**d**) and mother (**e**) of case 13

**Fig. 6:**
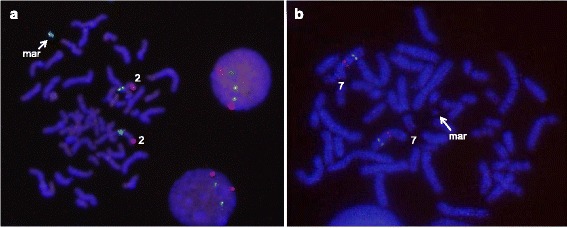
Identification of the chromosomal origin of the sSMC(2) by FISH in case 13. FISH analysis with MYCN (2p24,3; spectrum red) probe and LAF4 (2q11.2; spectrum green) probe shows a green hybridization signal on the marker chromosome, indicating that it is a derivative of chromosome 2 (**a**). No red signal was observable on any structure other than normal homologues of chromosome 7 using FISH analysis with the probes of ELN (7q11.23; spectrum red) and D7S485/D7S522 (7q31; spectrum green) (**b**)

**Fig. 7:**
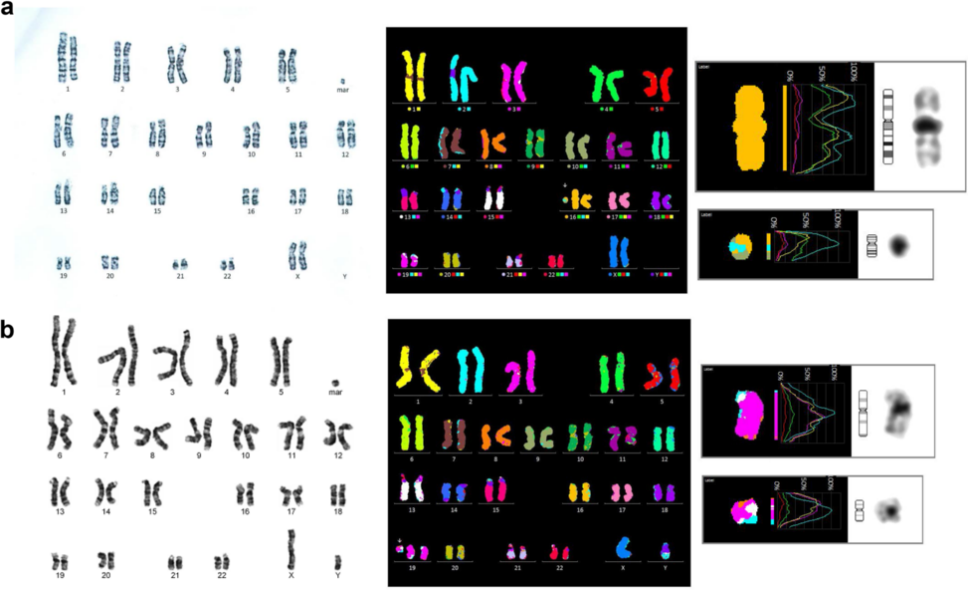
Two sSMCs were characterized using multicolor FISH (M-FISH), which demonstrated the presence of additional material that originated from chromosome 16 (**a**, case 10) and 19 (**b**, case 11)

For the remaining five marker chromosomes, M-FISH and/or array CGH identified the chromosomal origin. Two cases with a marker originating from chromosome 2 were characterized by array CGH. Array CGH detected a maternally inherited gain of 12.6 Mb derived from chromosome 2q11.1q12.3 (chr2: 95,529,039-108,083,956) with a log_2_ratio of 0.4890 and a de novo 5.7 Mb loss of chromosome 18p11.32p11.31 (chr18: 142,096-5,853,122) with a log_2_ ratio of -0.8833 in case 12 (Fig. 5a-b). A de novo 9.8 Mb gain of chromosome 2q11.1q12.1 (chr2: 95,529,039-105,358,887) with a log_2_ ratio of 0.3365 and maternally inherited 1.4 Mb gain of chromosome 7q11.23 (chr7: 72,726,578-74,139,390) with a log_2_ ratio of 0.4710 were identified in case 13 (Fig. 5c-e). Further FISH analysis confirmed that the 2q-amplified region, detected by array CGH, was localized to the marker chromosomes (Fig. 6). Three cases with low-level mosaicism were evaluated with M-FISH and the origin of the sSMCs was chromosome 6, 16 and 19, respectively (cases 9, 10 and 11) (Fig. 7).

**Fig. 8:**
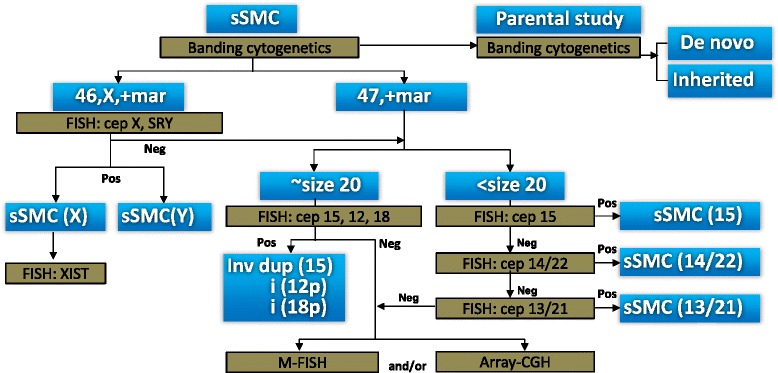
The algorithm for the determination of chromosomal origin of marker chromosomes

On the basis of our experience, the present knowledge of sSMC frequency [5] and the previously suggested characterization schemes [6–8], we have followed a modified algorithm that allowed the determination of the chromosomal origin of the marker chromosome in an effective manner using diverse techniques including banding cytogenetics, M-FISH and array CGH (Fig. 8).

## Discussion

In our study population, six (6/12, 50 %) marker chromosomes were derived from acrocentric autosomes, six were derived from non-acrocentric autosomes and one from the X chromosome. Marker chromosome derived from chromosome 15 was the most frequent sSMC identified in individuals with karyotype 47,XN,+mar (3/12, 25 %), similar to the reported frequency of 30 % in the literature [8]. In the present study, 2 sSMC(15)s were without euchromatin, but 1 sSMC(15) showed a maternally inherited euchromatic sSMC(15) spanning 15q11.1 to 15q13.1. Larger sSMC(15) with euchromatic content has been associated with a wide spectrum of clinical features from normal to full phenotype of the 15q11-q13 duplication syndrome (OMIM #608636) including autism, mental retardation, ataxia, seizures, developmental delays, and behavioral problems [9, 10], and generally, sSMC(15) spanning 15pter to 15q12 tend to have less severe phenotype than larger ones including 15pter to 15q14. While the identical large sSMC(15) was also identified in her phenotypically normal mother, the mother had a mosaic sSMC(15) (mos 47,XX,+mar[37]/46,XX[3]) while the proband had a single cell line, suggesting that the lack of mosaicism may be associated with the abnormal phenotype seen in the proband.

FISH analysis using centromeric probes for 14/22, confirmed three sSMCs as derived from chromosome 14 or 22. Following M-FISH analysis, two cases were identified as sSMC(14) and sSMC(22), respectively. However, the sSMC of case 8 was not resolved by M-FISH. This may be due to an underrepresentation of the region in M-FISH probes or a “flaring effect” of the fluorescence-intense centromeric signal [11]. The presence of a sSMC(14) is very rare [12] and among sSMC(14) cases with clinical signs, dysmorphic features and mental retardation are most often reported [8]. Regarding sSMC(22), 70 % of carriers are clinically normal [13] but distinct pathologic phenotypes, including CES (cat-eye syndrome, OMIM #115470) and ES (Emanuel syndrome, OMIM #609029) are associated with sSMC(22). The majority of CES is caused by bisatellited isodicentric marker chromosome containing CES critical region located in the most proximal 2-2.5 Mb of 22q11 [14] and ES is most often caused by a balanced translocation, t(11;22)(q23;q11.2), in one of the parents. Case 7 had an extra dicentric chromosome 22, however, analysis for CES critical region could not be done due to the insufficient amount of specimen. Furthermore, we were unable to obtain detailed clinical information regarding the presence or absence of major CES phenotypes including ocular coloboma, anal atresia, and renal malformations.

Marker chromosomes derived from non-acrocentric autosomes comprise about 40 % of all markers among individuals with karyotype 47,XN,+mar, and the risk of an abnormal phenotype associated with non-acrocentric autosomes is approximately 28 % [8, 15, 16]. In our study, five cases (5/12, 42 %) were derived from non-acrocentric autosomes and had clinical features of developmental delay or short stature, with the exception of case 9 for whom phenotypic information was not available. Two ring-shaped marker chromosomes were characterized by array CGH as originating from chromosome 2q. The majority of previously reported sSMC(2)s are ring-shaped sSMCs, as in this study, and a correlation of 2p11.2 with the presence of clinical abnormalities, and 2q11.2 with an absence of clinical signs have been suggested [17]. For case 12, we have assumed that the sSMC(2) was maternal in origin so the sSMC itself is harmless. However, for case 13, in addition to the sSMC(2), a duplication of 7q11.23 was identified by array CGH, and the genotype-phenotype correlation of sSMC(2) was not evident for this case. Marker chromosomes derived from non-acrocentric autosomes such as chromosome 6, 16, and 19 are also very rare (0.6–1.4 %) and the phenotype associated with each marker is not well established [6].

Marker chromosomes are found in 7-16 % of patients with TS and the marker chromosome is mainly from sex chromosomes, and only rarely from autosomes [18]. Screening of Y chromosome material in sSMC of TS patients is important because of its associated risk of gonadoblastoma [19]. Also, when sSMC is derived from X chromosome in TS patients, depending on the size and content of the sSMC(X)s, lack of the *XIST* locus (Xq13.2) may be associated with a more severe phenotype that includes mental retardation [18]. Although we did not perform FISH for the *XIST* gene, case 1 had no mental retardation, suggesting that her sSMC(X) contained *XIST*.

The extra derivative chromosome produced by the exchange of genomic material between two or more chromosomes is also very rare, except in ES. As shown in our case, parental karyotyping becomes relevant in tracing the origins of sSMCs. To our knowledge, this is the first reported case associating derivative marker chromosome involving chromosome 12 and 18. This patient with trisomy 12p and trisomy 18p showed incomplete cleft palate, developmental coordination disorder and development delay.

There are only a few well-established clinical syndromes associated with sSMCs originating from chromosomes 12, 15, 18 and 22 [20]. And the clinical outcome of the majority of marker chromosomes is highly variable, depending on their origin, size, euchromatin content, co-occurrence of uniparental disomy, and prevalence of aneuploidy in mosaic cases [21]. Generally, while there is no discernibly increased risk for fetal abnormalities if the marker has been inherited from a phenotypically normal parent, the risk for an abnormal phenotype in prenatally ascertained de novo cases is given as ~13 % [22]. Therefore the clinical management and genetic counseling depend on the characteristics of marker chromosomes and parental origin. In this regard, molecular cytogenetics, in combination with banding cytogenetics can provide precise information of the breakpoints of the marker chromosomes and accurate delineation of chromosomal content. The algorithm followed in this study proved as a straightforward and efficient strategy that can be used in most diagnostic molecular cytogenetic laboratories for characterization of sSMCs. Using this algorithm acrocentric sSMCs can be characterized in 2 days, and non-acrocentric sSMCs requiring M-FISH or array CGH can be characterized in 5 days.

## Conclusion

This report is the largest study on sSMCs in Korea and expands the spectrum of sSMCs that are molecularly characterized. The stepwise application of molecular cytogenetic methods proved as both practical and efficient strategy that allowed straightforward and accurate characterization of sSMCs. And an accurate identification of the genetic content of sSMCs should provide more information on genotype-phenotype correlation and for genetic counseling.

## Abbreviations

array CGH, array comparative genomic hybridization; CES, cat-eye syndrome; ES, Emanuel syndrome; FISH, fluorescence *in situ* hybridization; ISCN 2013, international system for human cytogenetic nomenclature (2013); M-FISH, whole chromosome painting multicolor-FISH; OMIM, online mendelian inheritance in man; sSMCs, small supernumerary marker chromosomes; TS, Turner syndrome

## Additional file

Additional file 1: Table S1. The panel of FISH probes used for identification of marker chromosomes. (DOCX 17 kb)
