# Supplementary material for: Identification of small marker chromosomes using microarray comparative genomic hybridization and multicolor fluorescent in situ hybridization
Source: Mol Cytogenet. 2016 Aug 8;9:61. doi: 10.1186/s13039-016-0273-5 (PMC4977864; doi:10.1186/s13039-016-0273-5)
Supplement: Additional file 1: Table S1. — The panel of FISH probes used for identification of marker chromosomes. (DOCX 17 kb) [file 13039_2016_273_MOESM1_ESM.docx]

| Additional file 1: Table S1. The panel of FISH probes used for identification of marker chromosomes | | | |
| --- | --- | --- | --- |
| Chromosome | Region | Locus | Manufacturer |
| 2 | 2p24.3/2q11.2 | MYCN/LAF4 | Cytocell |
| 6 | 6p11.1-q11 Alpha Satellite DNA | D6Z1 | Vysis |
| 7 | 7q11.23/7q31 | ELN/D7S486, D7S522 | Vysis |
| 12 | Whole chromosome | - | MetaSystems |
| 13, 21 | Alpha Satellite | D13Z1/D21Z1 | Cytocell |
| 14, 22 | Alpha Satellite | D14Z1/D22Z1 | Cytocell |
| 14 | Whole chromosome | - | MetaSystems |
| 15 | 15p11.2 Satellite III DNA | D15Z1 | Vysis |
| 15 | 15q11-q13 | D15S11 | Vysis |
| 15 | Whole chromosome | - | MetaSystems |
| 16 | Whole chromosome | - | MetaSystems |
| 18 | Whole chromosome | - | MetaSystems |
| 19 | Whole chromosome | - | MetaSystems |
| 22 | Whole chromosome | - | MetaSystems |
| X | Xp11.1-q11.1 Alpha Satellite DNA | DXZ1 | Cytocell |
| Y | Yp11.31 | SRY | Cytocell |
